# Supplementary material for: Metabolic stress-induced cardiomyopathy is caused by mitochondrial dysfunction due to attenuated Erk5 signaling
Source: Nat Commun. 2017 Sep 8;8:494. doi: 10.1038/s41467-017-00664-8 (PMC5591279; doi:10.1038/s41467-017-00664-8)
Supplement: Supplementary file 1 — Supplementary Information [file 41467_2017_664_MOESM1_ESM.pdf]

Description of Supplementary Files

File Name: Supplementary Information
Description: Supplementary Figures.

File Name: Peer Review File

Supplementary information

Supplementary Figure 1

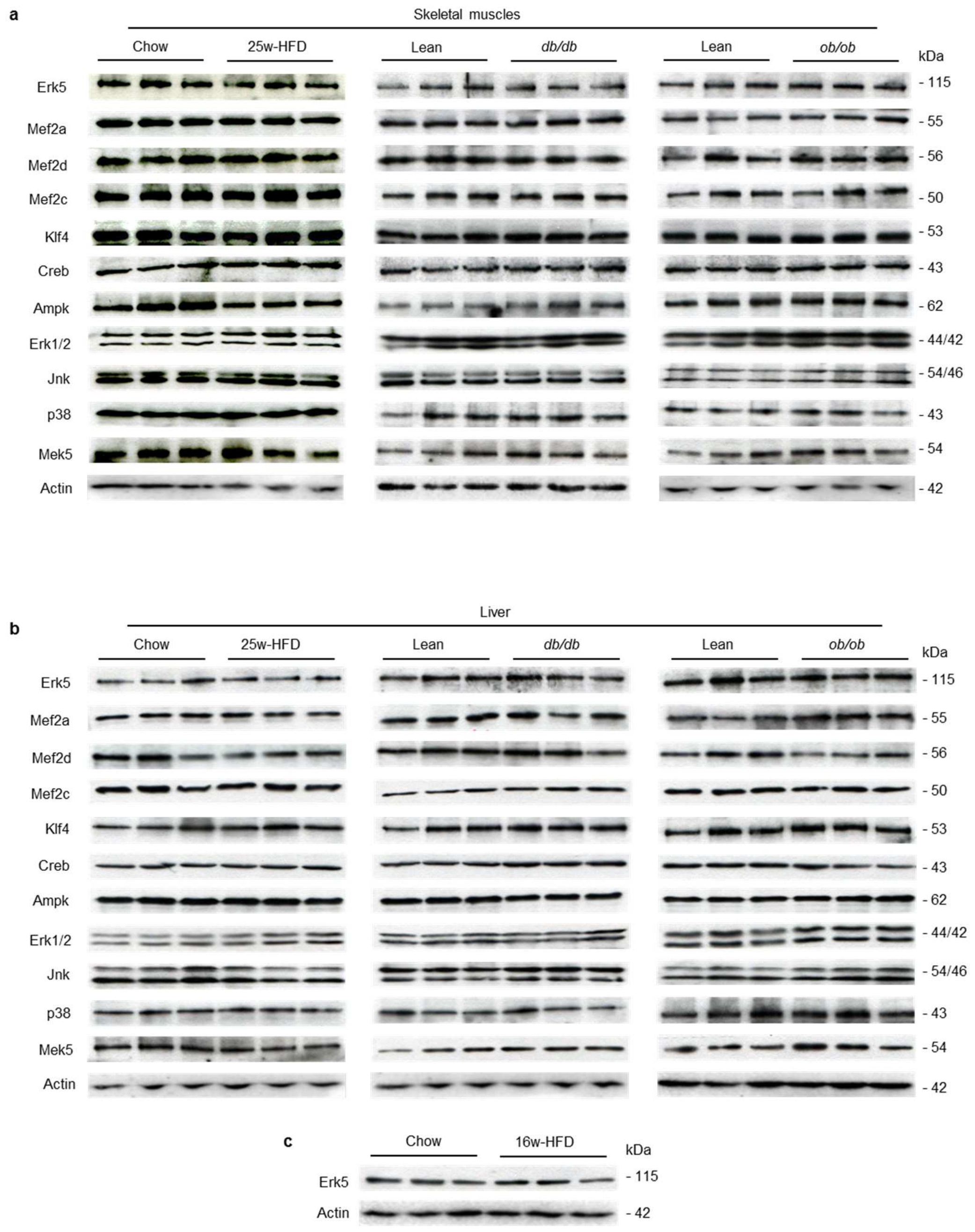

Supplementary Figure 1. Expression screening by immunoblot analyses. Expression of Erk5, Mef2a, Mef2d, Mef2c, Klf4, Creb, Ampk, Erk1/2, Jnk, p38, and Mek5 was comparable in the obesity or diabetic skeletal muscles (**a**) and liver (**b**) to control group. (**c**) The expression of Erk5 was at a similar level in the hearts of C57BL/6J mice fed with HFD for 16 weeks, compared with 16-week chow fed mice. Actin expression is the protein loading control.

Supplementary Figure 2

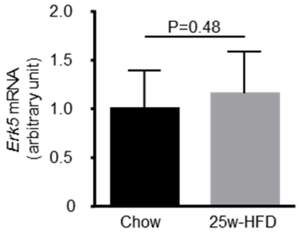

Supplementary Figure 2. qPCR analysis showed no reduction in mRNA expression of *Erk5* in the hearts from C57BL/6J mice with 25-week HFD. n=6 independent experiments. Data are presented as means ± SD.

Supplementary Figure 3

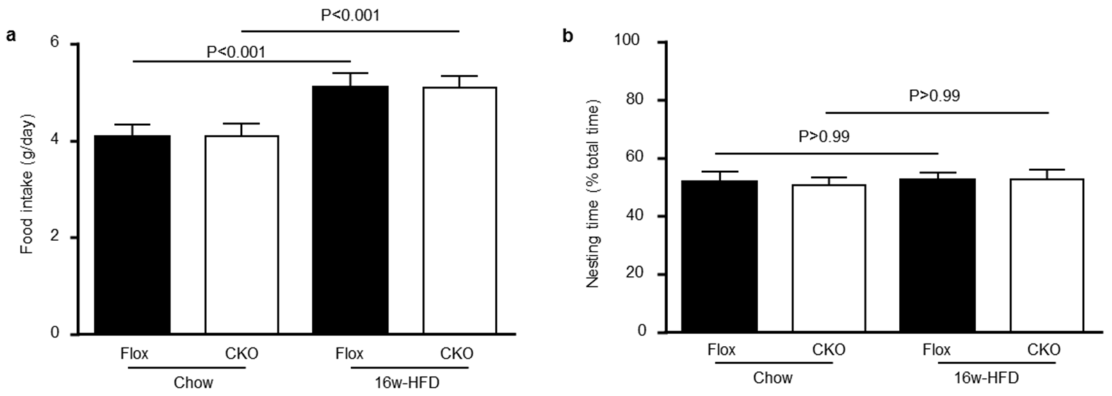

Supplementary Figure 3. Behaviour analyses of Flox and CKO in response to chow diet or HFD. (**a**) Food intake was increased in HFD fed groups. (**b**) Nesting time was comparable among experimental groups. n=5 mice per group. Data are presented as means ± SD.

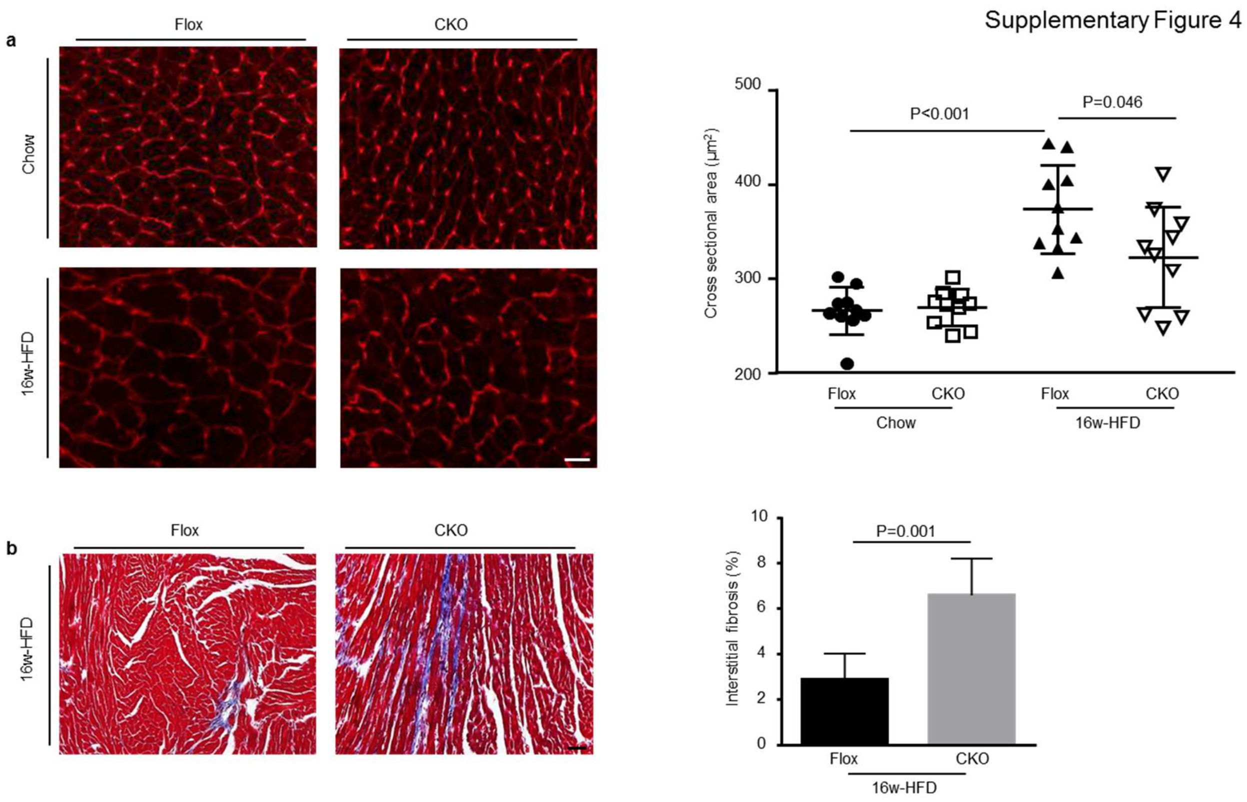

Supplementary Figure 4. Morphological changes in the Flox and CKO hearts after diet stress. (**a**) Wheat germ agglutinin (WGA) staining of heart cross-sections (left panel, scale bar: 20μm). The measurement of cross-sectional area demonstrated enlarged cardiomyocytes in response to HFD for 16 weeks (right panel). n=10 mice per group. (**b**) Masson's trichrome staining detected more interstitial fibrosis HFD-CKO hearts (left image, scale bar: 50μm). Quantification of the fibrosis area is shown in the bar graphs (right panel). n=6 mice per group. Data are presented as means ± SD.

Supplementary Figure 5

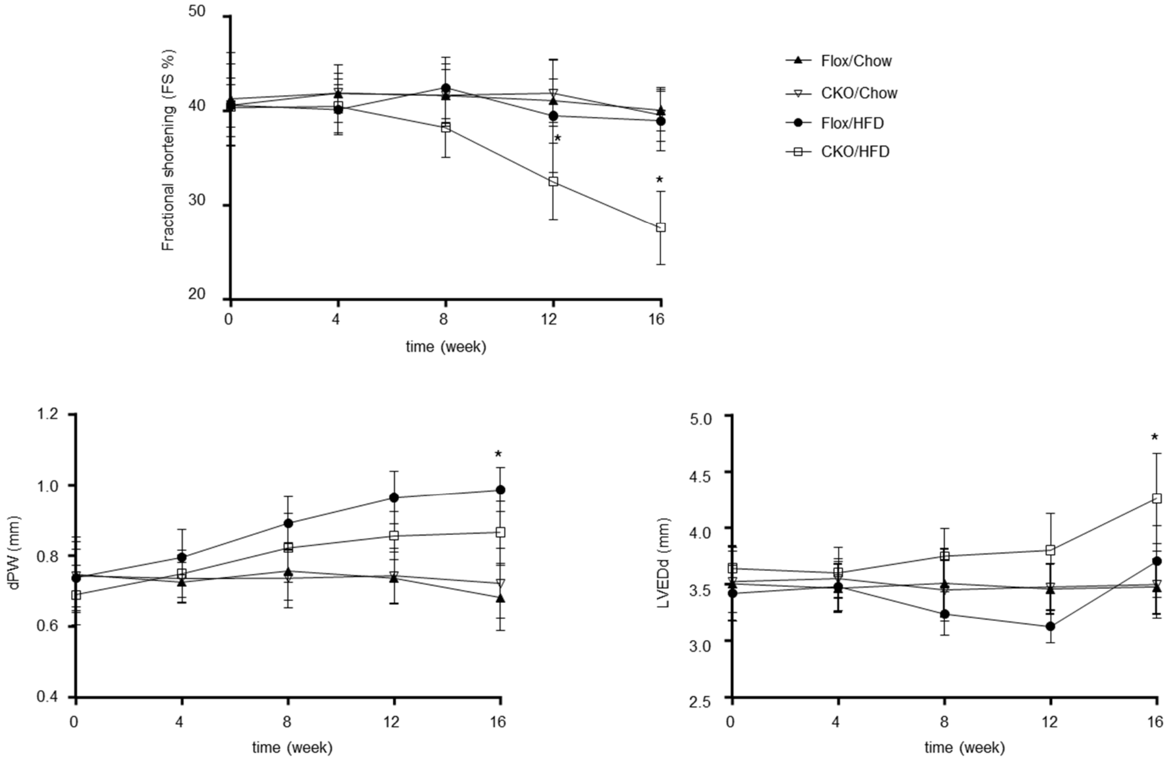

Supplementary Figure 5. Cardiac function of Flox and CKO mice fed with chow or HFD was measured and demonstrated by FS%, thickness of diastolic posterior wall (dPW), and left ventricular end diastolic dimension (LVEDd) at 4-week intervals. Data are presented as means ± SD (*P<0.05, vs chow groups, n=7 mice per group).

Supplementary Figure 6

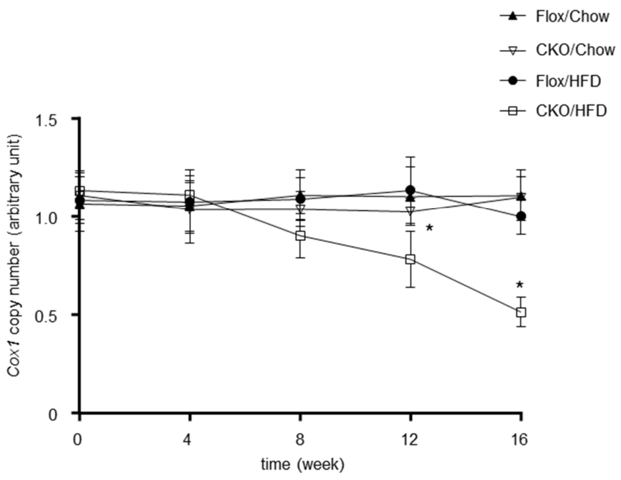

Supplementary Figure 6. The content of mtDNA in Flox and CKO hearts was measured by the ratio of Cox1 to cyclophilin A at 4-week intervals. Data are presented as means ± SD (*$P<0.05$, vs chow groups, n=7 mice per group).

Supplementary Figure 7

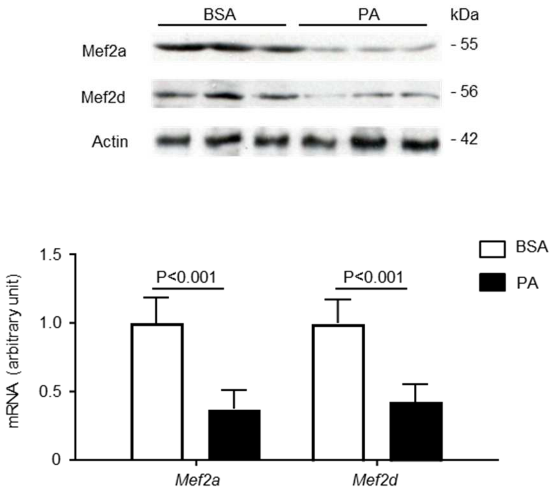

Supplementary Figure 7. Mef2a and Mef2d were decreased by the treatment of palmitate acid (PA). Immunoblot analysis (upper panel) and qPCR (lower panel) confirmed the decreased protein and mRNA levels of Mef2a or Mef2d in adult rat cardiomyocytes (ARCMs) treated with PA (500µM, 8 hours). Actin expression is the protein loading control. n=6 independent experiments per group. Data are presented as means ± SD.

Supplementary Figure 8

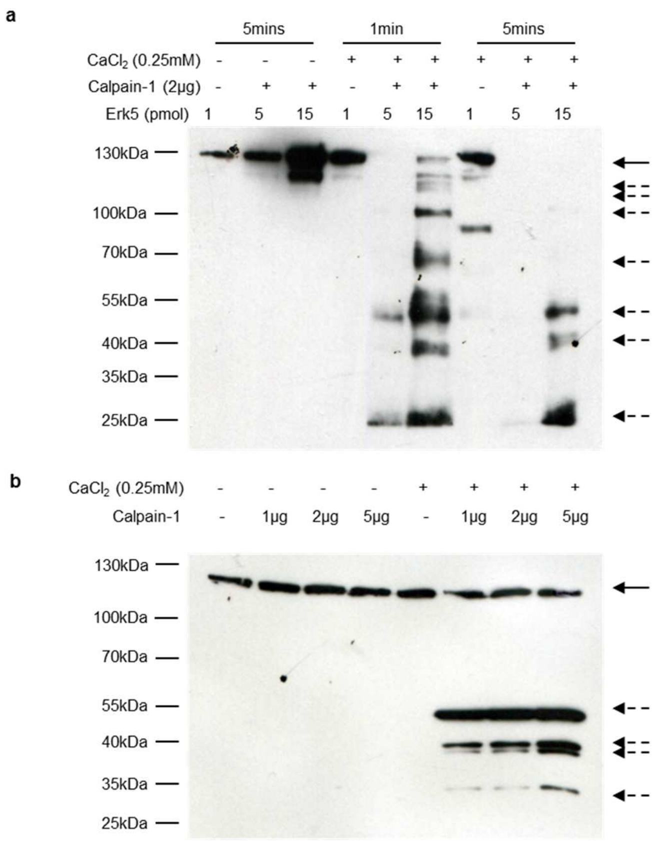

Supplementary Figure 8. Erk5 was degraded by calpain-1 in the presence of Ca^{2+}. (**a**) A cell-free system was used by incubation of human recombinant calpain-1 (2µg) with 5pmol or 15pmol human recombinant Erk5 for 1 minute or 5 minutes. The cleavage reaction was analyzed by immunoblotting using anti-Erk5 antibody. (**b**) COS-7 cells were transfected with flag-tagged human Erk5. Flag-Erk5 was immunoprecipitated prior to the incubation with human recombinant calpain-1 at 37°C for 30 minutes. Multiple bands ranging from 55KD to 25KD were captured by immunoblotting using anti-Flag antibody, and band density was increased on a calpain-1 dose-dependent manner. Arrow showing for a full length of Erk5, dashed arrows for cleaved fragments.

Supplementary Figure 9

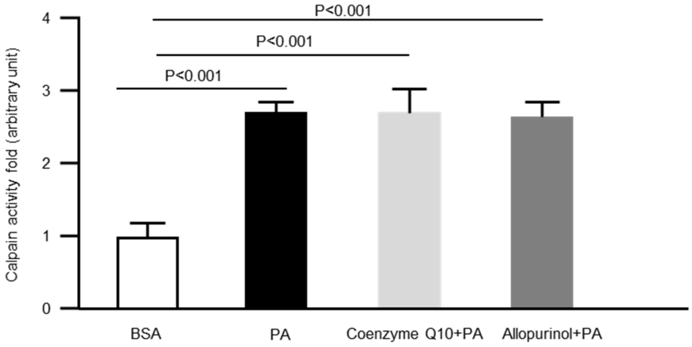

Supplementary Figure 9. Mitochondria-targeted antioxidant (coenzyme Q10, 200μM, 2 hours) or xanthine oxidase inhibitor (Allopurinol, 200μM, 2 hours) did not prevent the increased calpain activity induced by PA in ARCMs. n=5 independent experiments per group. Data are presented as means ± SD.

Supplementary Figure 10

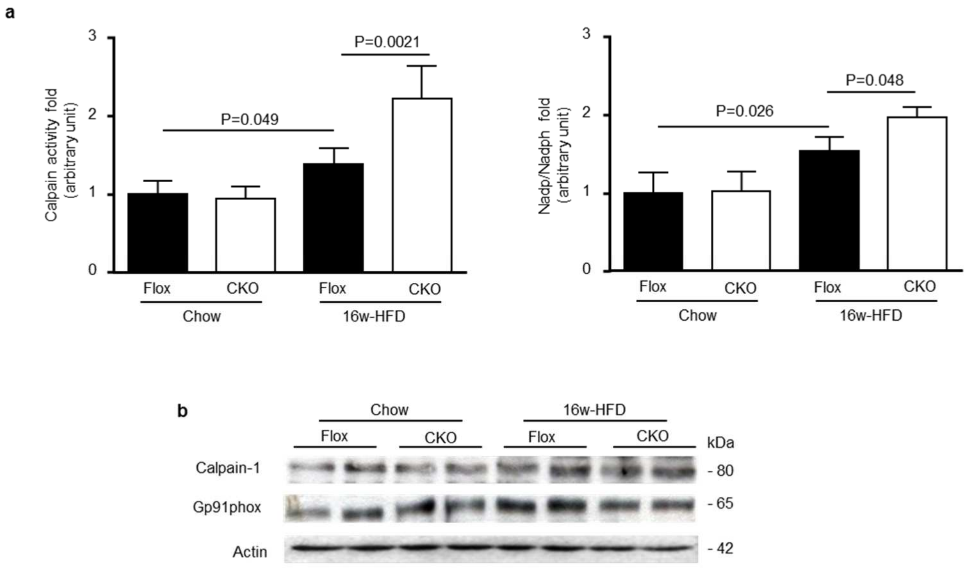

Supplementary Figure 10. Activity and expression of calpain and Nadph. (**a**) Increased calpain activity and Nadph oxidase activity were shown in HFD-CKO hearts, n=7 mice per group. Data are presented as means ± SD. (**b**) The protein expression of calpain-1 and gp91phox remained unchanged despite HFD in Flox and CKO mice. Actin is the protein loading control.

Supplementary Figure 11

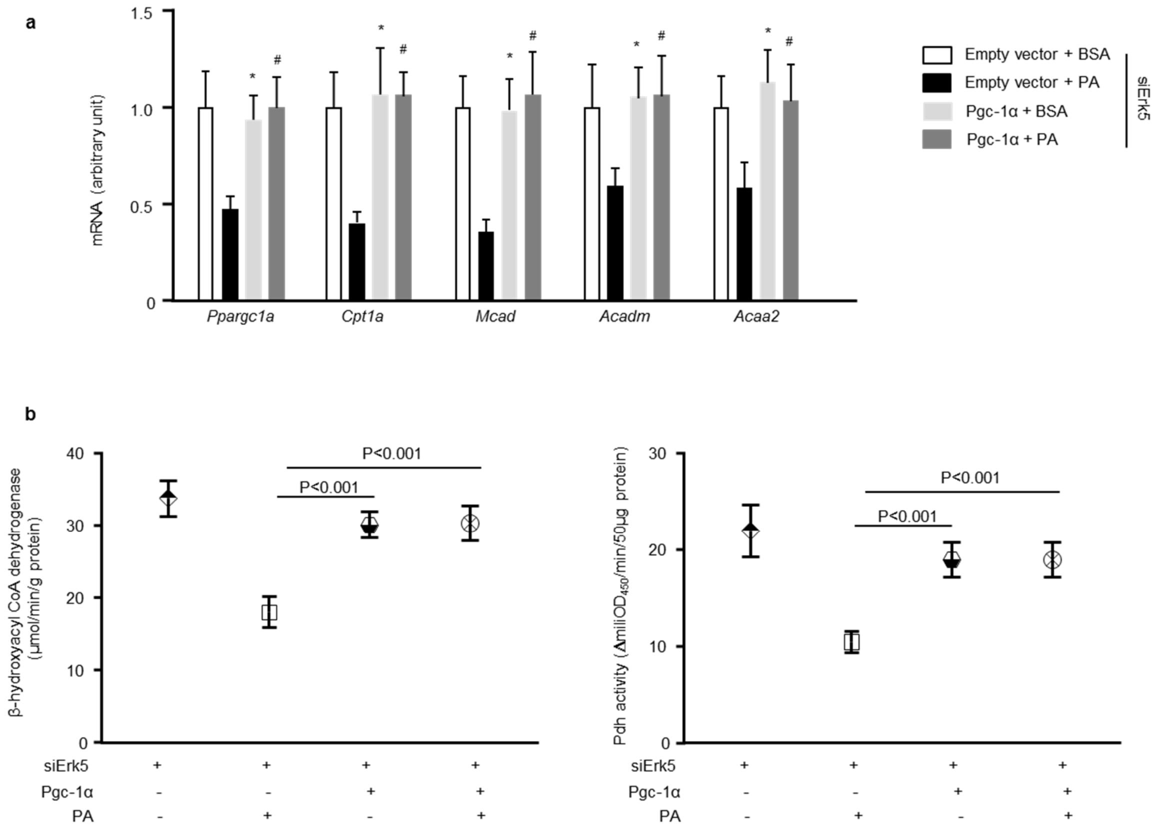

Supplementary Figure 11. Pgc-1α overexpression was able to alleviate mitochondrial dysfunction in Erk5-deficient ARCMs after PA stimulation. (**a**) Transcript levels of key mitochondrial genes were up-regulated by Pgc-1α overexpression in Erk5-deficient ARCMs following PA treatment. (**b**) Mitochondrial fuel oxidation measured by the activity of β-hydroxylacyl CoA dehydrogenase and pyruvate dehydrogenase (Pdh) was improved following Pgc-1α overexpression. n=5 independent experiments per group. Data are presented as means ± SD.

Supplementary Figure 12

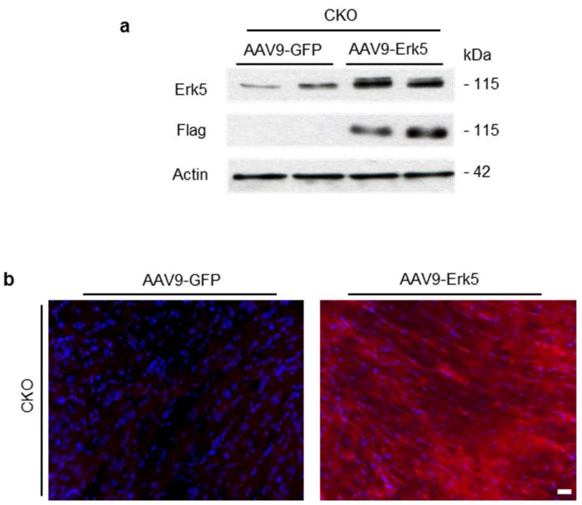

Supplementary Figure 12. Evaluation of Erk5 expression in AAV9-Erk5 virus injected mice. (**a**) Immunoblot analyses showed substantial Erk5 expression in CKO mice having AAV9-Erk5 injection. Actin is the protein loading control. (**b**) Immunohistochemistry using anti-Flag antibody showed ectopic Erk5 expression in CKO hearts after AAV9-Erk5 virus injection. DAPI visualized their nuclei (scale bar: 20μm).

Supplementary Figure 13

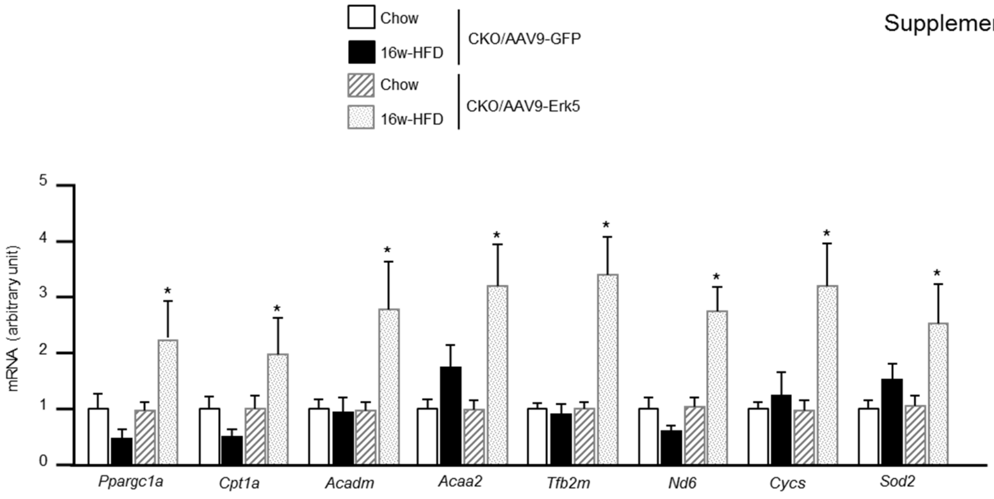

Supplementary Figure 13. Transcript levels of key mitochondrial genes were up-regulated along with Erk5 restoration in AAV9-Erk5 injected CKO mice subject to 16-week HFD. Data are presented as means ± SD. * P<0.05, vs CKO/AAV9-GFP group, n=6 mice per group.

Supplementary Figure 14

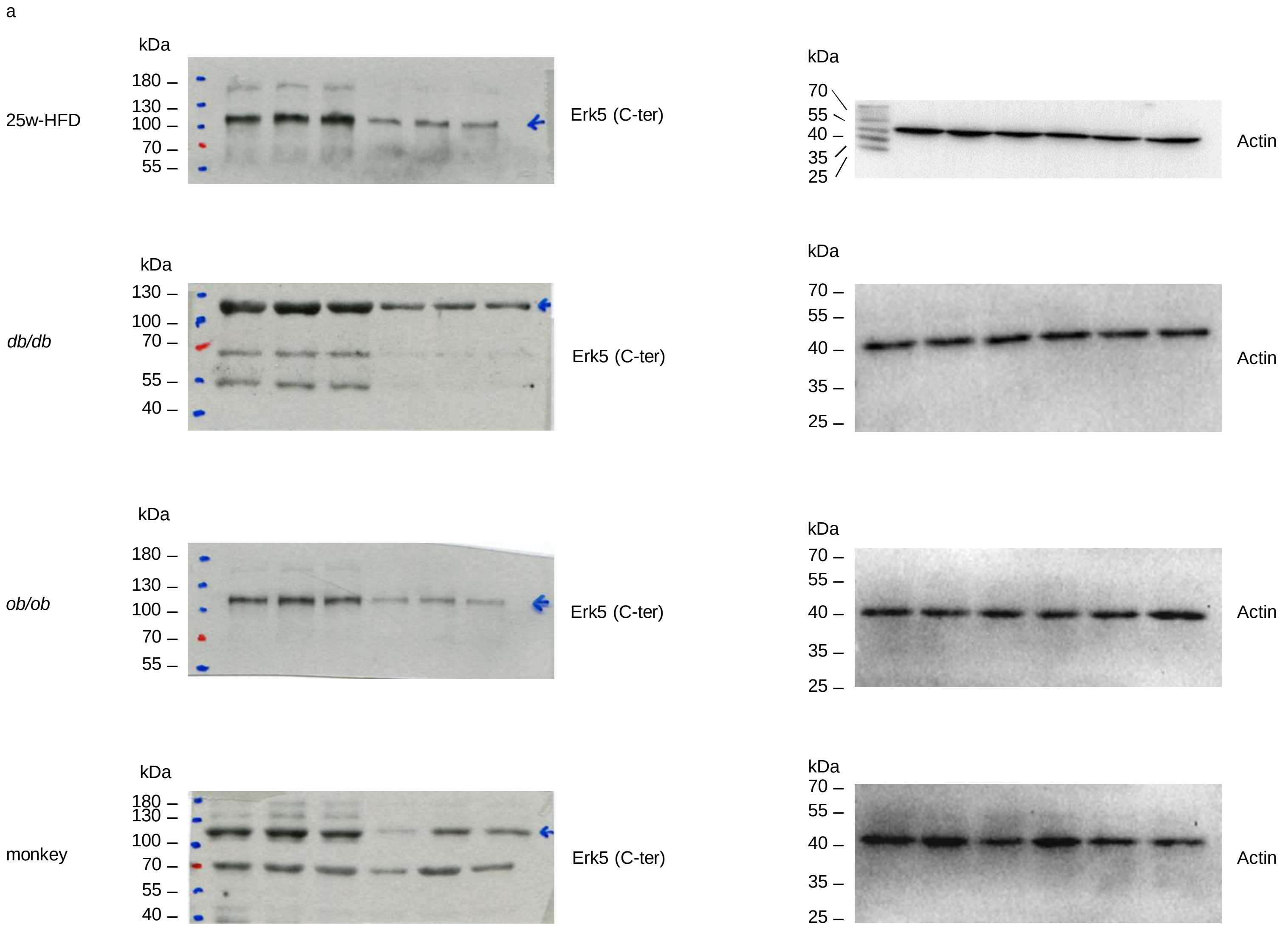

b

Heart extracts from Chow/ 25-HFD mice

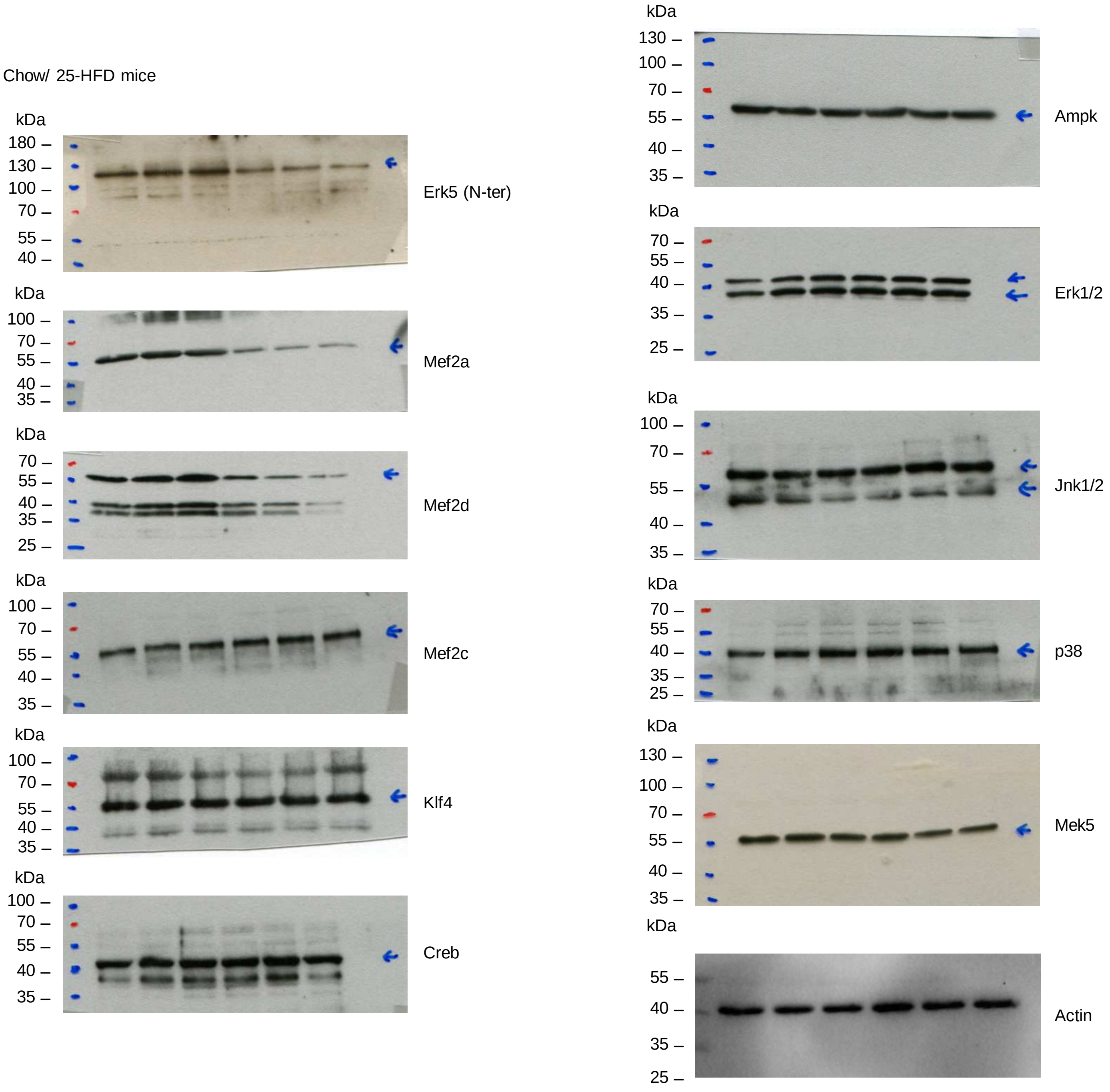

c

Heart extracts from Lean or db/db mice

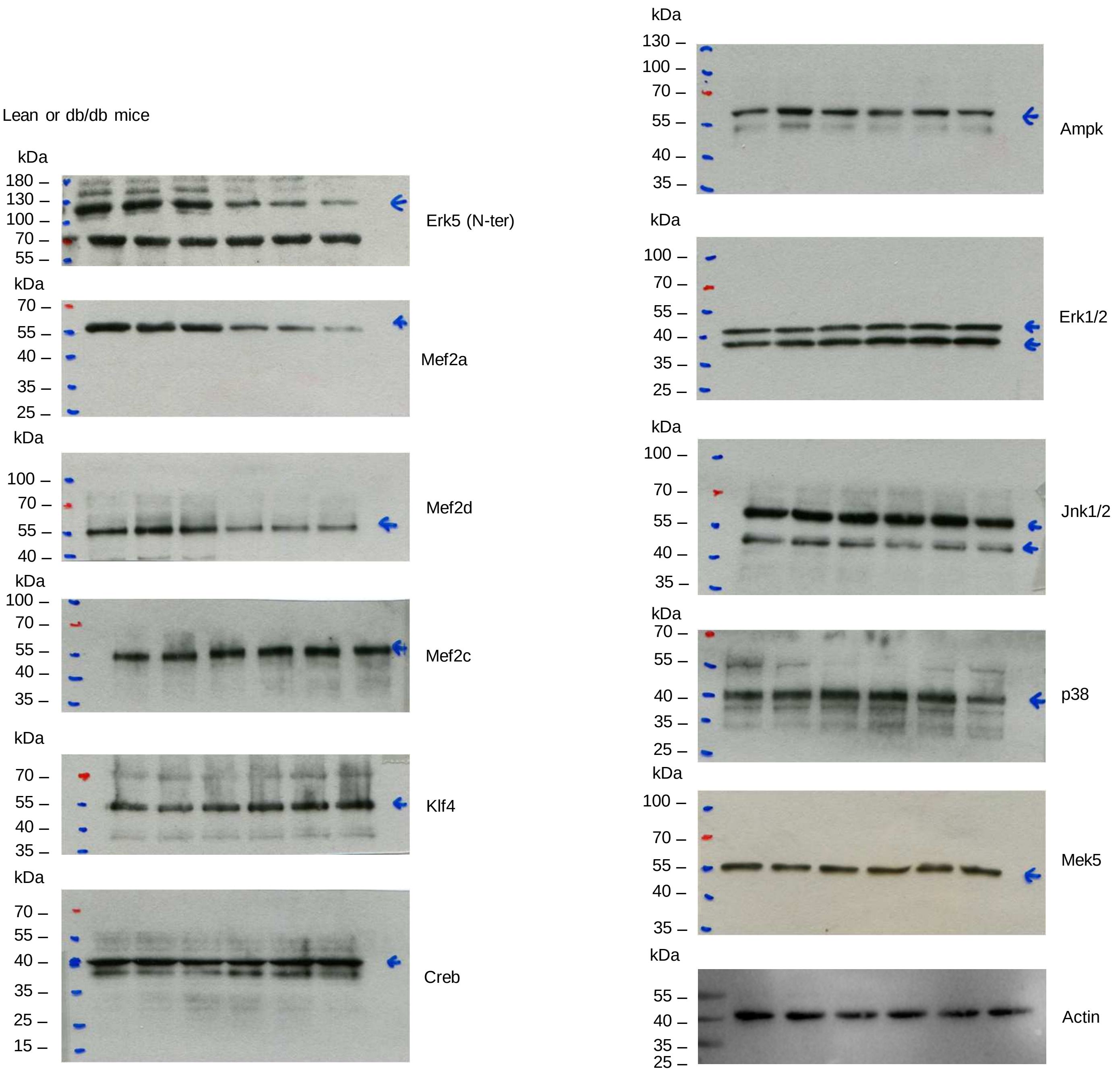

d

Heart extracts from Lean or ob/ob mice

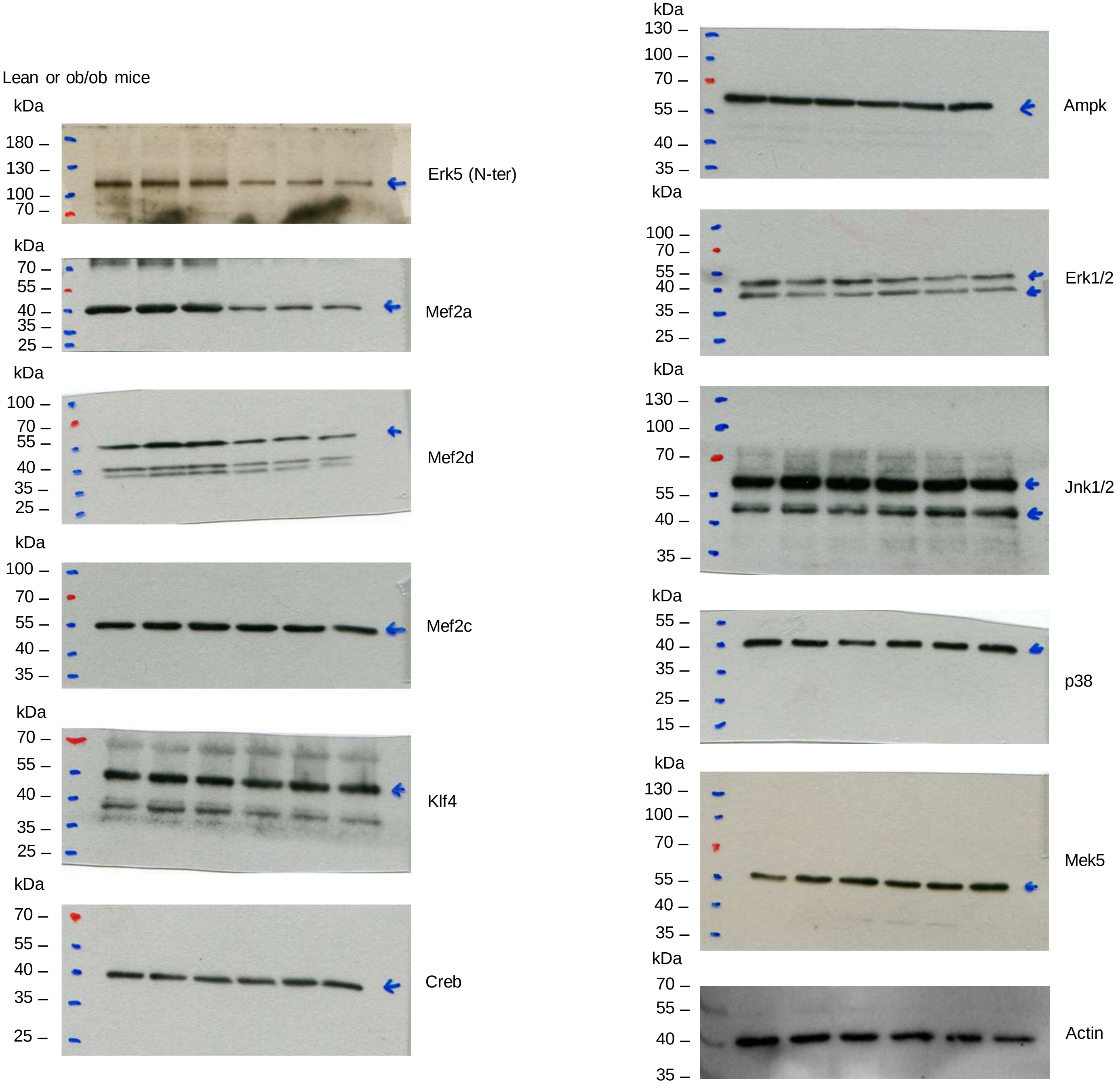

e

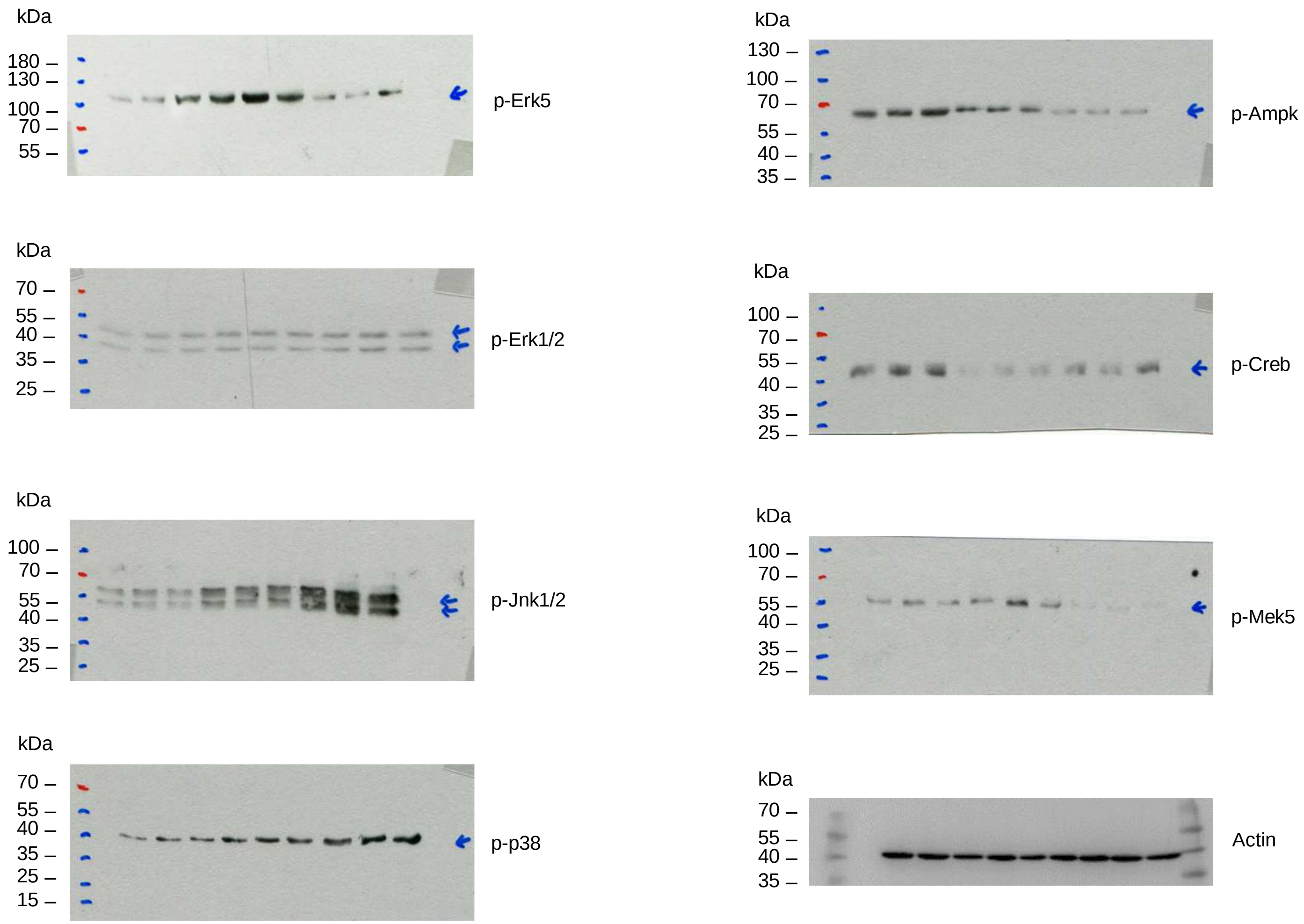
kDa
180
130
100
70
55
p-Erk5
kDa
70
55
40
35
25
p-Erk1/2
kDa
100
70
55
40
35
25
p-Jnk1/2
kDa
70
55
40
35
25
15
p-p38
kDa
130
100
70
55
40
35
p-Ampk
kDa
100
70
55
40
35
25
p-Creb
kDa
100
70
55
40
35
25
p-Mek5
kDa
70
55
40
35
Actin

f

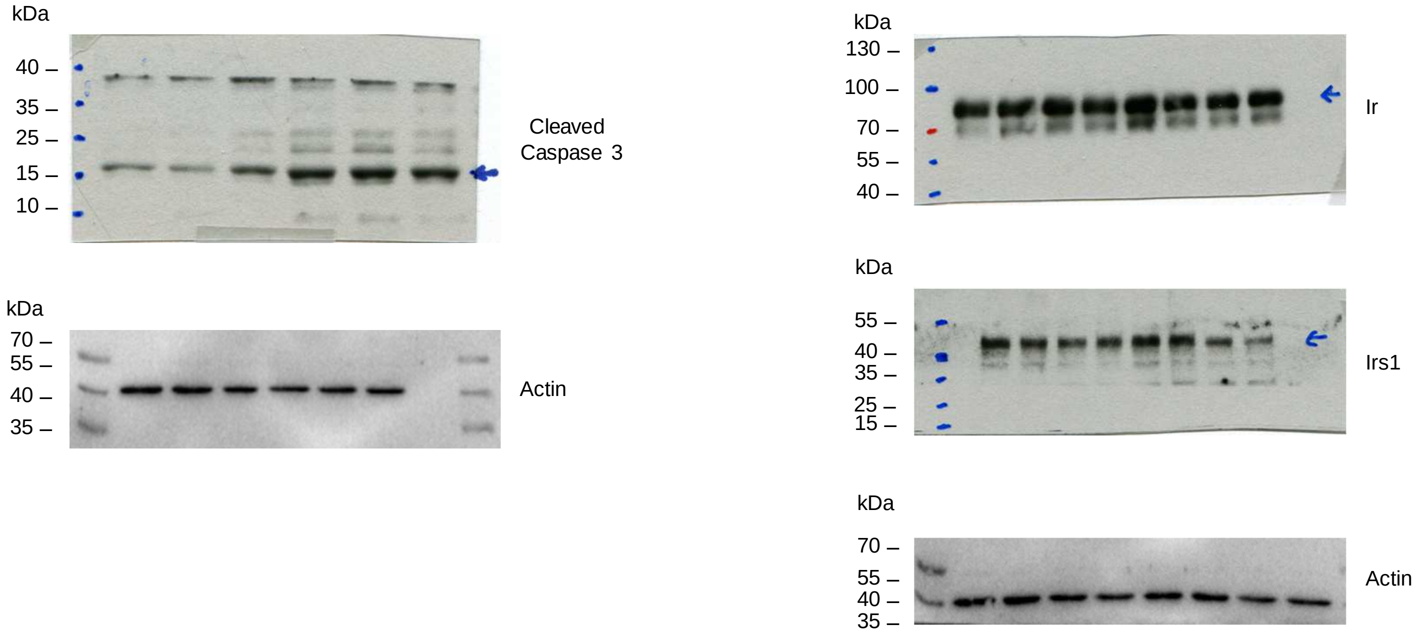
kDa
40
35
25
15
10
Cleaved Caspase 3
kDa
70
55
40
35
Actin
kDa
130
100
70
55
40
Ir
kDa
55
40
35
25
15
Irs1
kDa
70
55
40
35
Actin

g

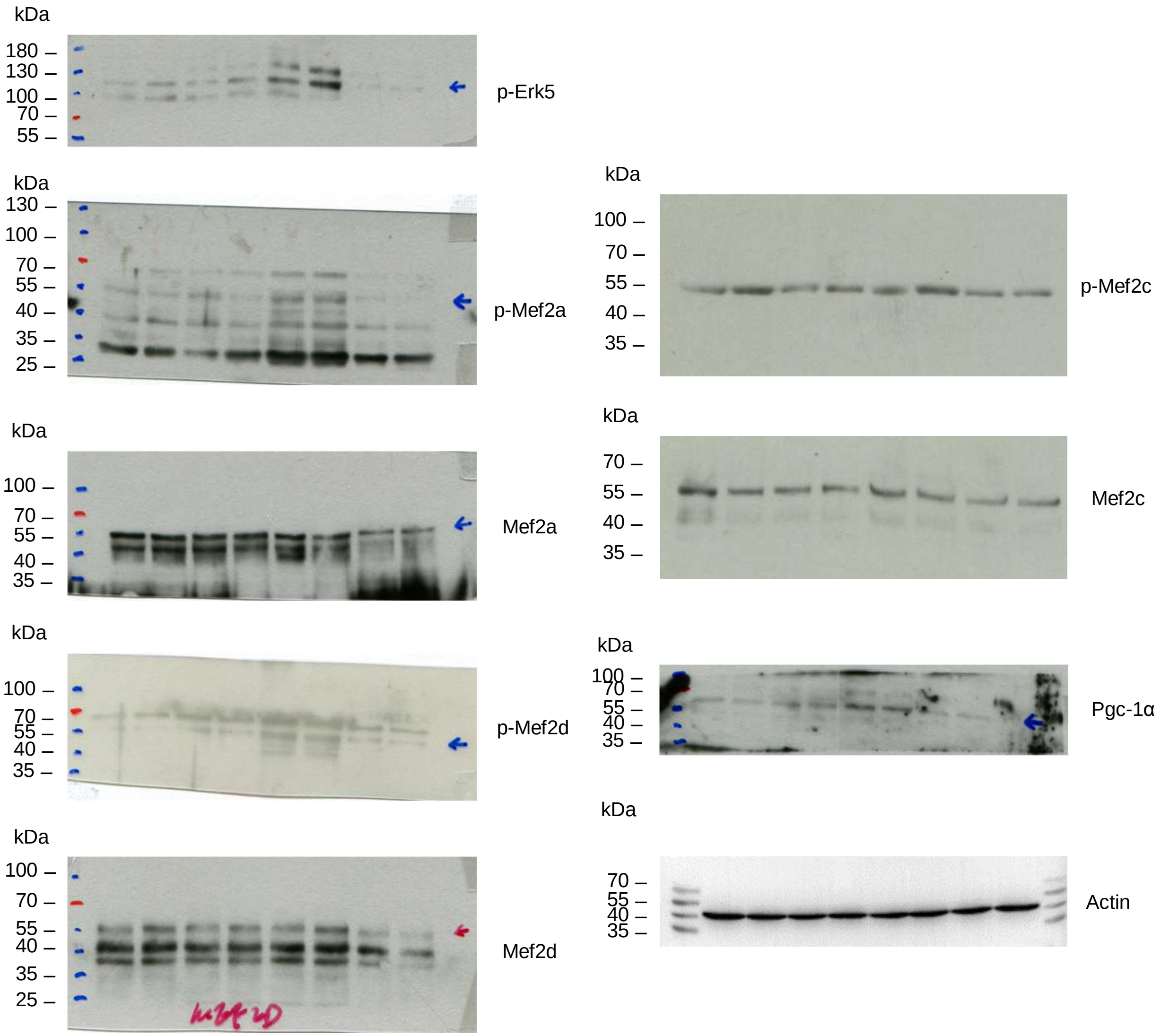
kDa
180 –
130 –
100 –
70 –
55 –
p-Erk5
130 –
100 –
70 –
55 –
40 –
35 –
25 –
p-Mef2a
Mef2a
35 –
p-Mef2d
Mef2d
p-Mef2c
Mef2c
Pgc-1α
Actin

h

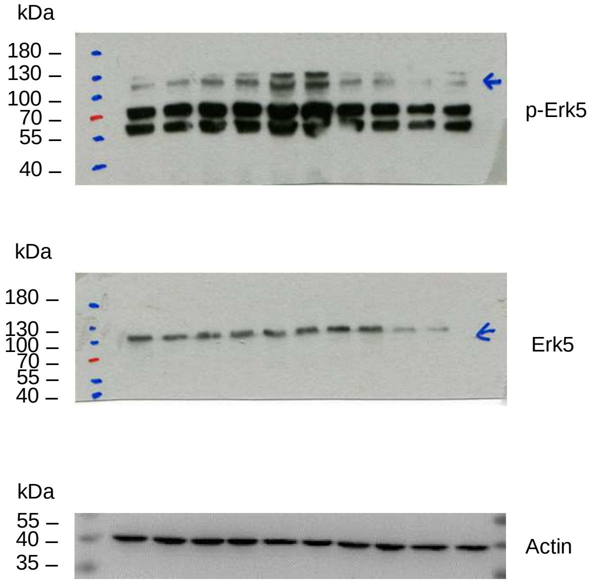
kDa
180 –
130 –
100 –
70 –
55 –
40 –
35 –
p-Erk5
Erk5
Actin

i

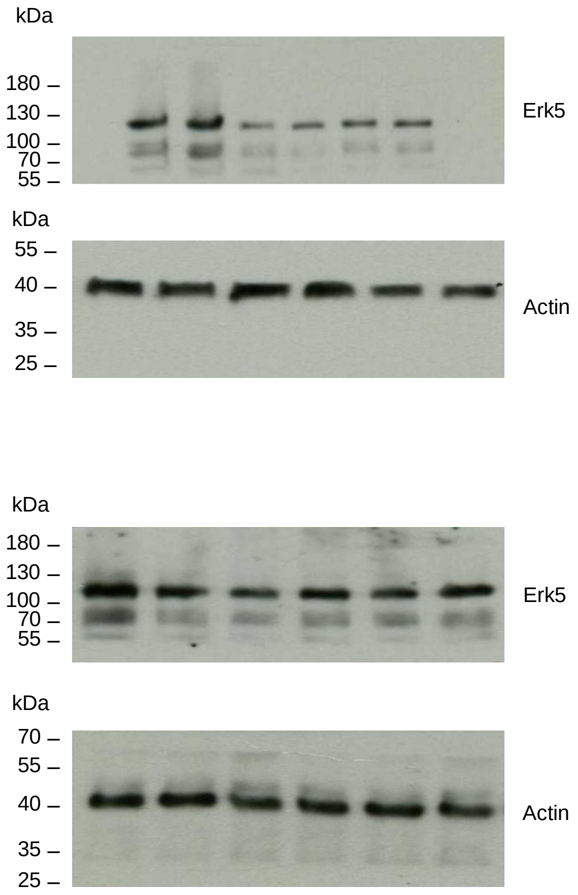
kDa
180
130
100
70
55
Erk5
kDa
55
40
35
25
Actin
kDa
180
130
100
70
55
Erk5
kDa
70
55
40
35
25
Actin

j

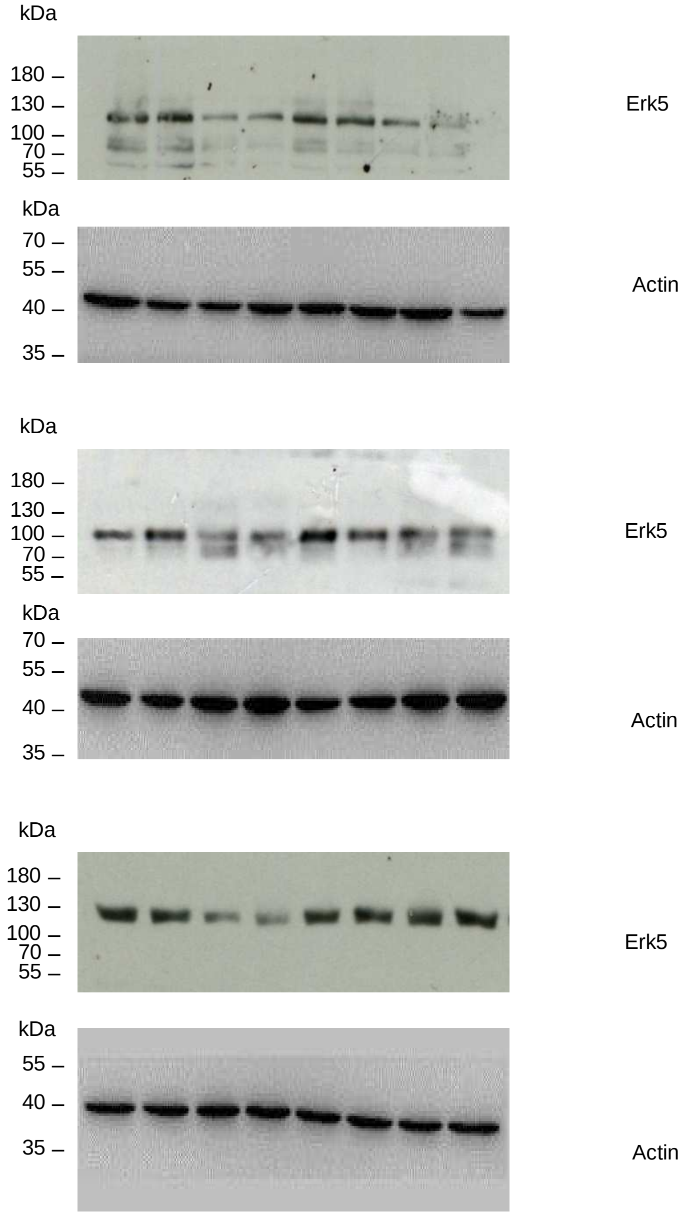
kDa
180
130
100
70
55
Erk5
kDa
70
55
40
35
Actin
kDa
180
130
100
70
55
Erk5
kDa
70
55
40
35
Actin
kDa
180
130
100
70
55
Erk5
kDa
55
40
35
Actin

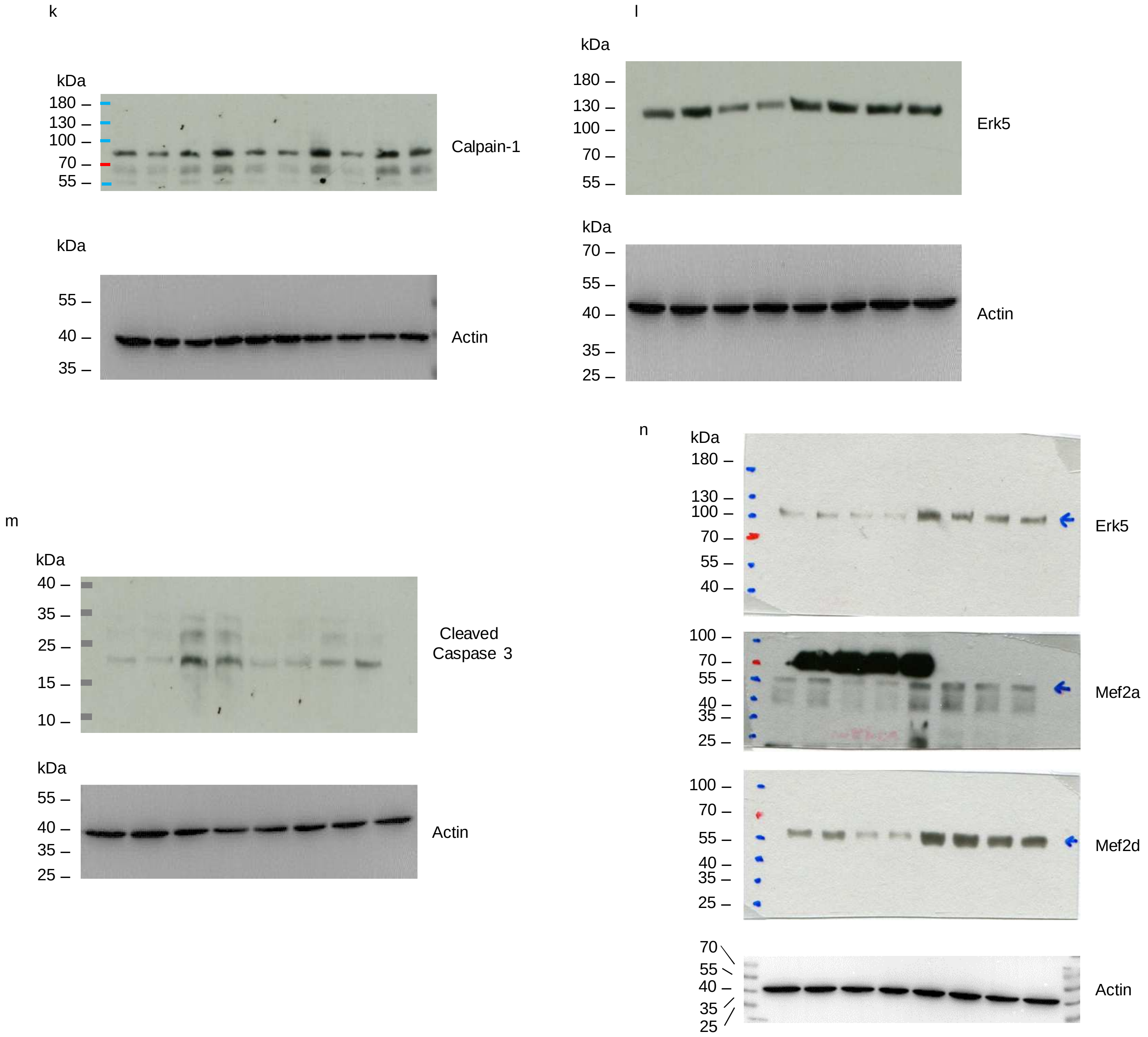
k
kDa
180 –
130 –
100 –
70 –
55 –
Calpain-1
kDa
55 –
40 –
35 –
Actin
l
kDa
180 –
130 –
100 –
70 –
55 –
Erk5
kDa
70 –
55 –
40 –
35 –
25 –
Actin
m
kDa
40 –
35 –
25 –
15 –
10 –
Cleaved Caspase 3
kDa
55 –
40 –
35 –
25 –
Actin
n
kDa
180 –
130 –
100 –
70 –
55 –
40 –
Erk5
100 –
70 –
55 –
40 –
35 –
25 –
Mef2a
100 –
70 –
55 –
40 –
35 –
25 –
Mef2d
70
55
40 –
35
25
Actin

o

Skeletal muscles from Chow/ 25-HFD mice

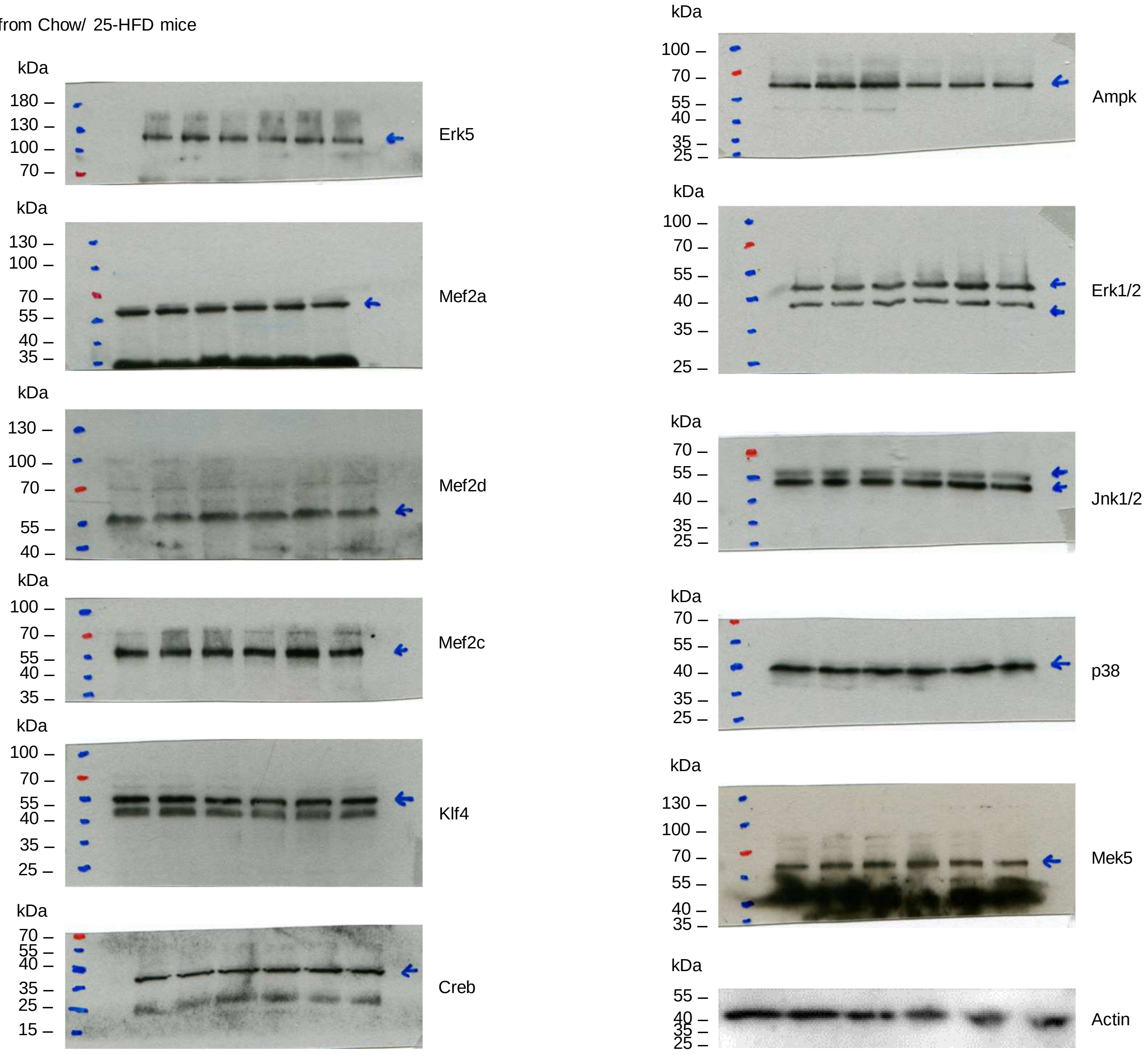

p

Skeletal muscles from Lean or db/db mice

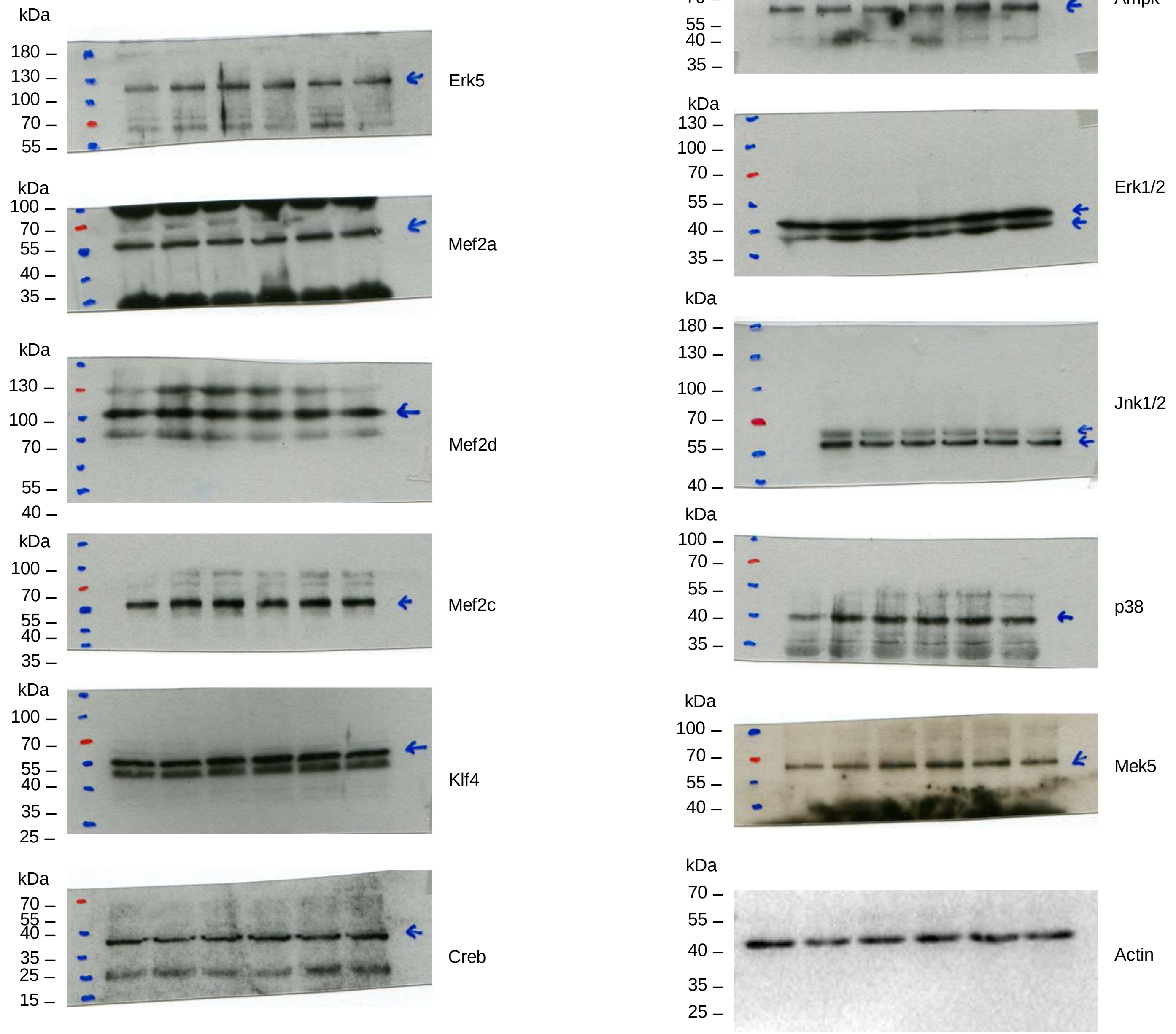

q

Skeletal muscles from Lean or ob/ob mice

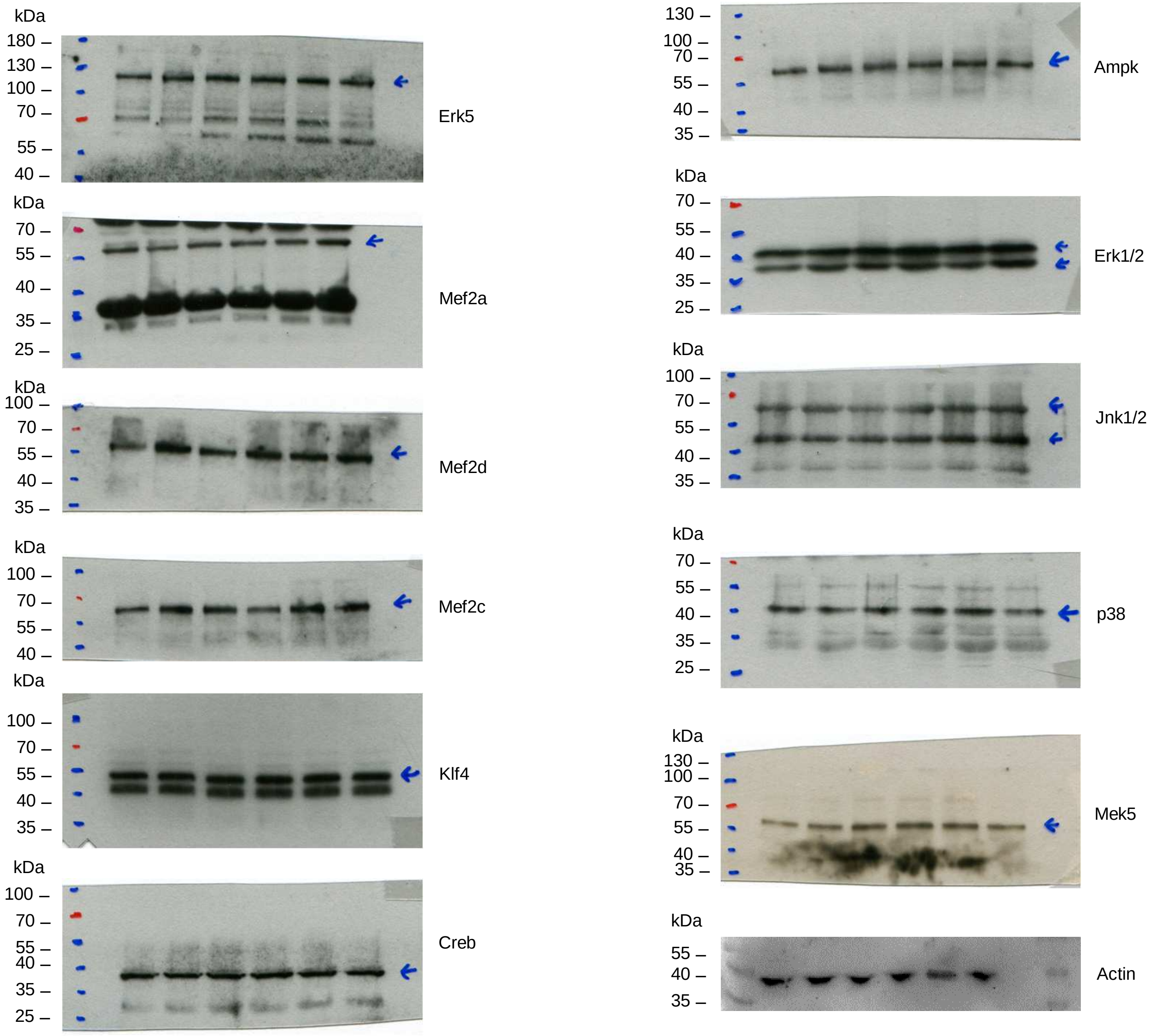

r

Liver from Chow/ 25-HFD mice

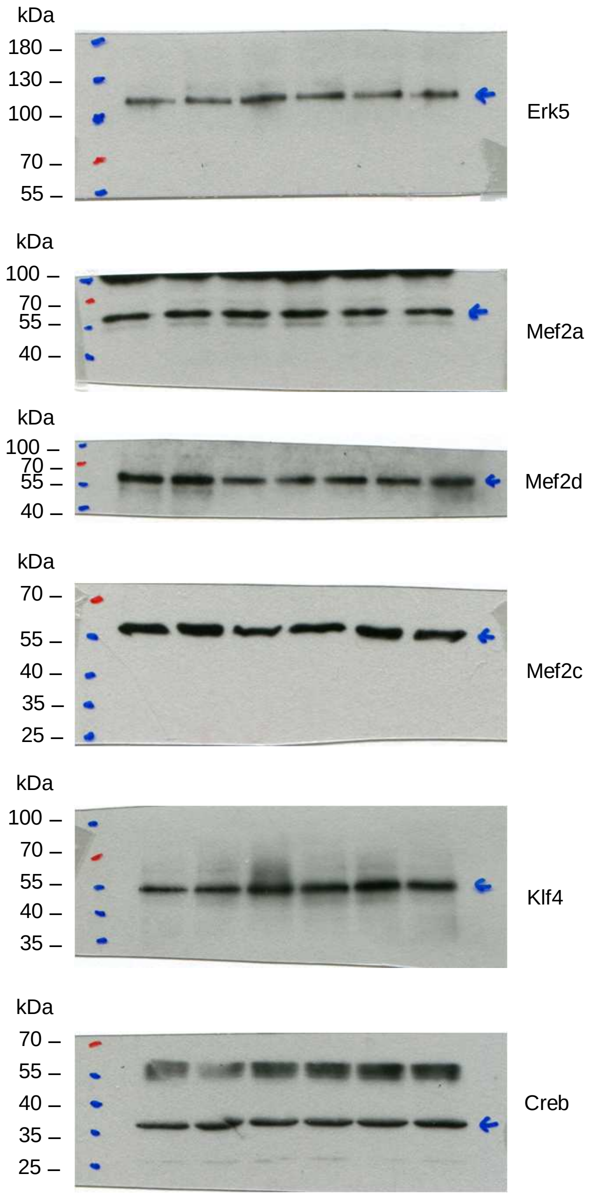

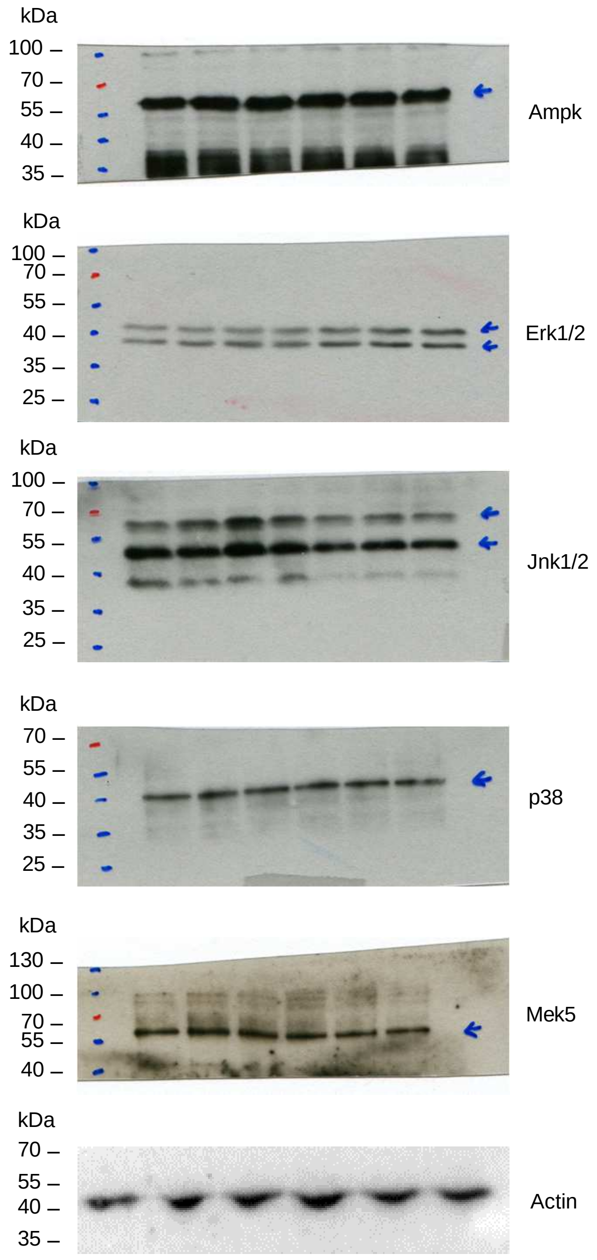

s

Liver from Lean or db/db mice

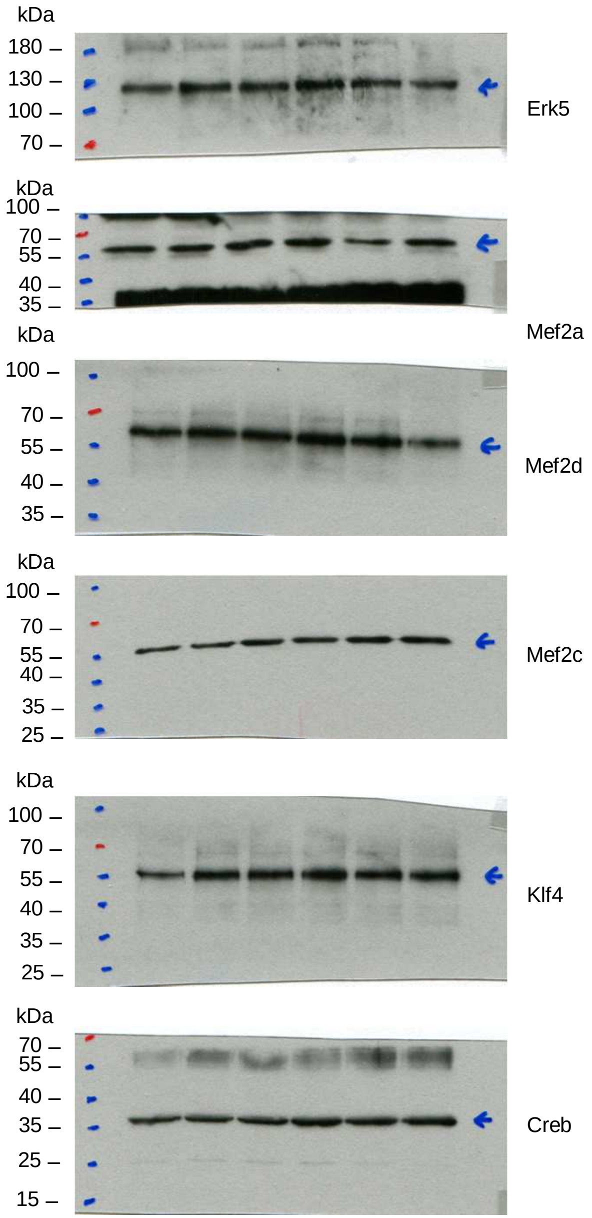

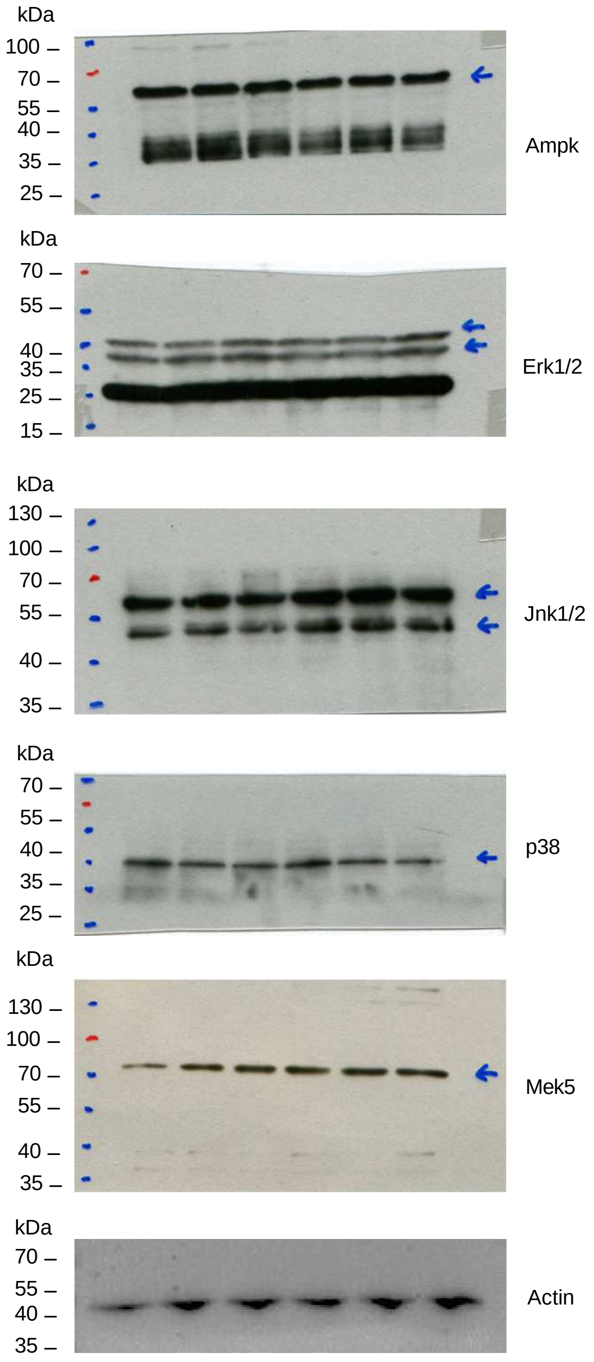

t

Liver from Lean or ob/ob mice

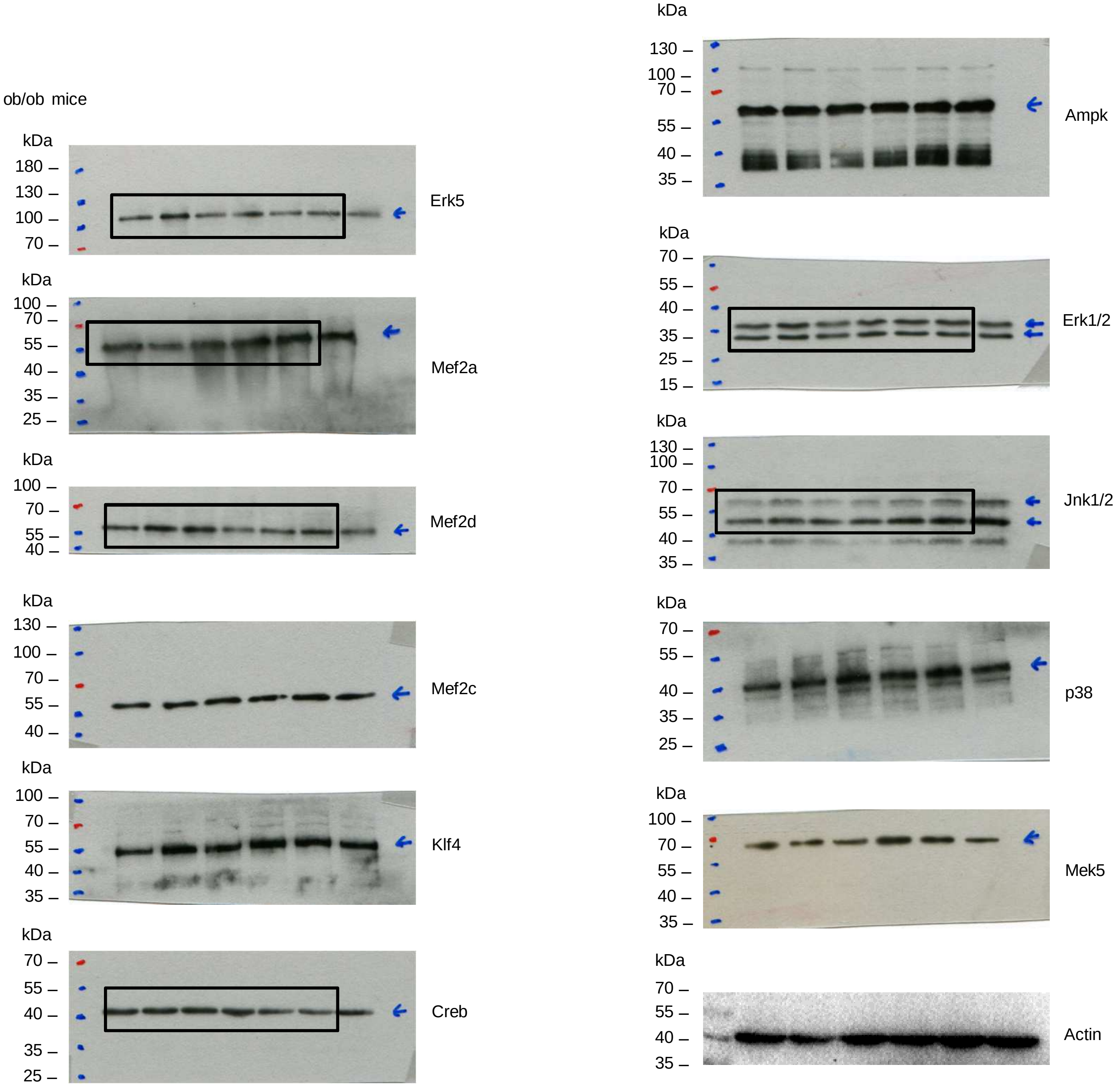

u

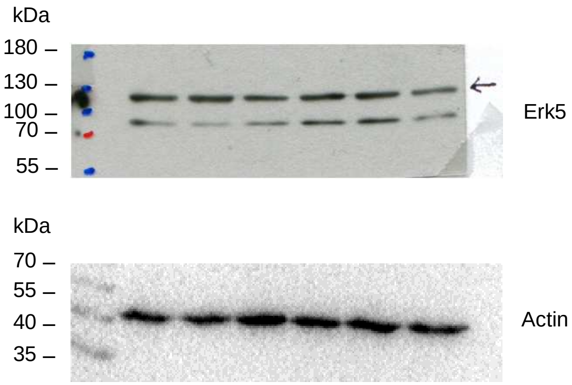
kDa
180 –
130 –
100 –
70 –
55 –
Erk5
kDa
70 –
55 –
40 –
35 –
Actin

v

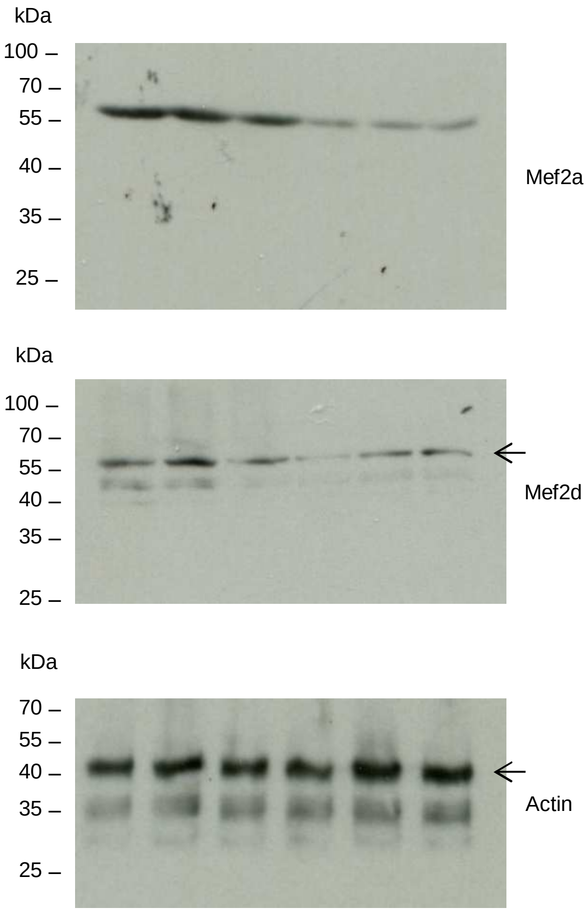
kDa
100 –
70 –
55 –
40 –
35 –
25 –
Mef2a
kDa
100 –
70 –
55 –
40 –
35 –
25 –
Mef2d
kDa
70 –
55 –
40 –
35 –
25 –
Actin

w

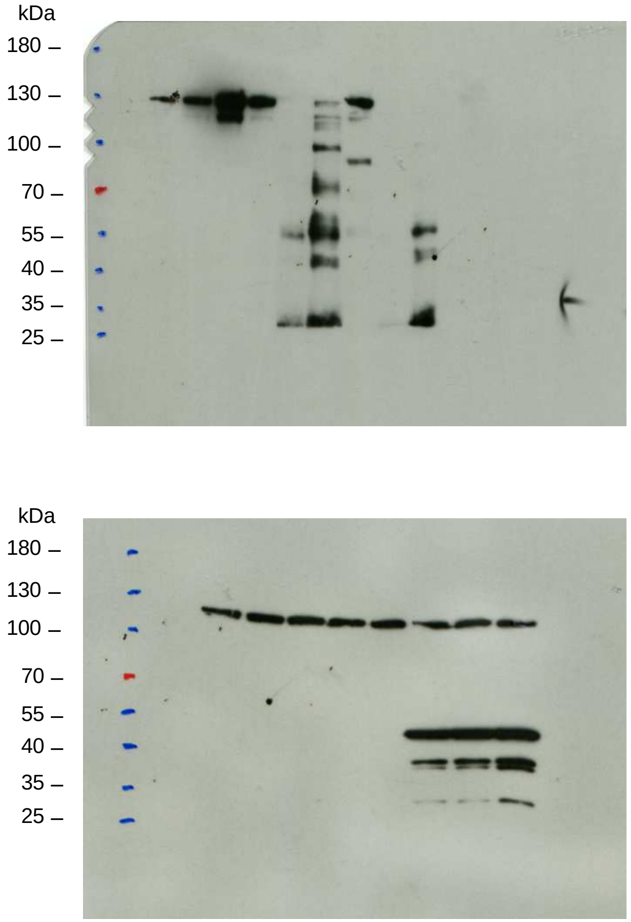

kDa
180
130
100
70
55
40
35
25
kDa
180
130
100
70
55
40
35
25

x

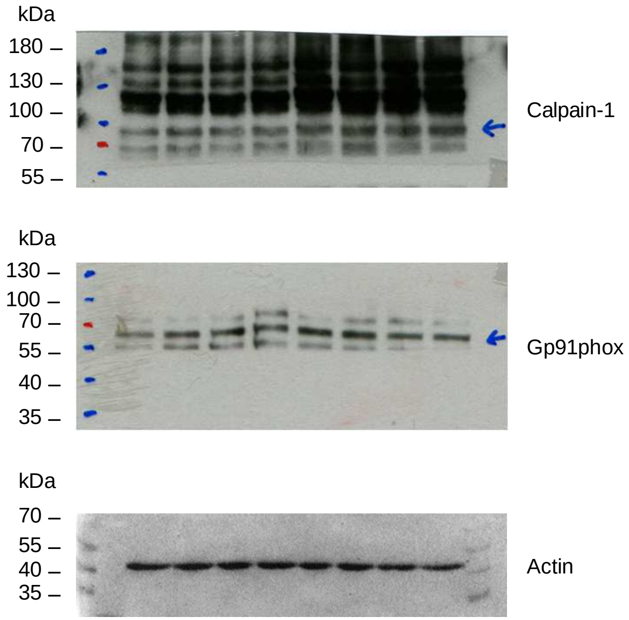

kDa
180
130
100
70
55
Calpain-1
kDa
130
100
70
55
40
35
Gp91phox
kDa
70
55
40
35
Actin

y

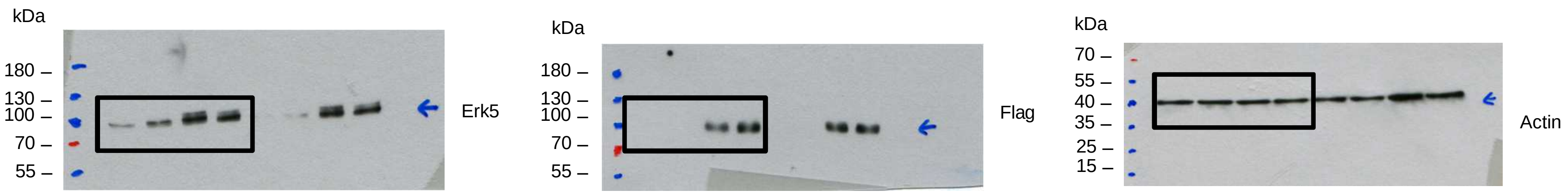

Supplementary Figure 14. Full scan images of Western blots. (**a**) Membranes from Fig. 1a. (**b**) Membrane from Fig. 1b left panel. (**c**) Membranes from Fig. 1b middle panel. (**d**) Membranes from Fig. 1b right panel. (**e**) Membranes from Fig. 1c. (**f**) Membranes from Fig. 4. (**g**) Membranes from Fig. 6a. (**h**) Membranes from Fig. 6b. (**i**) Membranes from Fig. 6f. (**j**) Membranes from Fig. 6g. (**k**) Membranes from Fig. 6h. (**l**) Membranes from Fig. 6i. (**m**) Membranes from Fig. 7c. (**n**) Membranes from Fig. 8a. (**o**) Membranes from Supplementary Fig. 1a left panel. (**p**) Membranes from Supplementary Fig. 1a middle panel. (**q**) Membranes from Supplementary Fig. 1a right panel. (**r**) Membranes from Supplementary Fig. 1b left panel. (**s**) Membranes from Supplementary Fig. 1b middle panel. (**t**) Membranes from Supplementary Fig. 1b right panel. (**u**) Membranes from Supplementary Fig. 1c. (**v**) Membranes from Supplementary Fig. 7. (**w**) Membranes from Supplementary Fig. 8. (**x**) Membranes from Supplementary Fig. 10b. (**y**) Membranes from Supplementary Fig. 12. The parts of the membranes that are presented in the figures are boxed. The molecular weight ladder is Thermo Scientific PageRuler Prestained Protein Ladder (26616).
